# Case report: Page kidney with multiple serosal effusions caused by bilateral spontaneous renal subcapsular hemorrhage

**DOI:** 10.3389/fmed.2024.1290470

**Published:** 2024-01-24

**Authors:** Leibo Wang, Zuze Qiu, Shun Zhan, Guanyu Shi, Wei He, Zhuangding Cen, Feng Xu, Wu Tian, Daobing Li

**Affiliations:** ^1^Surgery, Guizhou Orthopaedic Hospital, Guiyang, China; ^2^Surgery, Beijing Jishuitan Hospital Guizhou Hospital, Guiyang, China; ^3^Department of Urology, Affiliated Hospital of Zunyi Medical University, Zunyi, China; ^4^Department of Urology, Fenggang County People's Hospital, Zunyi, China

**Keywords:** Page kidney, secondary hypertension, multiple serous cavity effusion, subcapsular hematoma, renal insufficiency

## Abstract

Page kidney is caused by the perirenal or subcapsular accumulation of blood or fluid pressing on the renal parenchyma and is a rare cause of secondary hypertension. In this study, we report a case of Page caused by bilateral spontaneous subcapsular renal hematoma, the main manifestations of which were secondary hypertension, multiple serous effusions, and renal insufficiency. After admission, drug blood pressure control was ineffective. After bilateral perirenal effusion puncture and drainage were performed to relieve bilateral perirenal compression, blood pressure gradually dropped to normal, multi-serous cavity effusion (pericardial, thoracic, and abdominal effusion) gradually disappeared, and kidney function returned to normal. Secondary hypertension caused by Page kidney can be treated. When Page kidney is complicated with multiple serous effusions, the effect of antihypertensive drugs alone is poor, and early perineal puncture drainage can achieve better clinical efficacy.

## Introduction

Page kidney is a rare urological disease in which perirenal or subcapsular blood or fluid accumulation compresses the renal parenchyma and triggers the activation of the renin–angiotensin–aldosterone axis, leading to systemic hypertension (1). In recent years, most reported cases of Page kidney are mainly caused by trauma and iatrogenic injury, which is related to blunt trauma of the kidney or invasive diagnosis and treatment operations in and around the kidney, and non-traumatic or spontaneous causes of cases are rare (1–4). Polyserous effusion is a common and difficult clinical disease that refers to the simultaneous effusion of two or more serous cavities (pericardial cavity, thoracic cavity, and abdominal cavity). Studies (5) have pointed out that malignant tumors, infectious diseases, and autoimmune diseases are the main causes, and approximately 38% of patients have unknown causes.

In this study, we report an unusual case of Page kidney with bilateral spontaneous subcapsular hematoma, characterized by secondary hypertension and polyserous effusion.

## Case presentation

**Figure 1:**
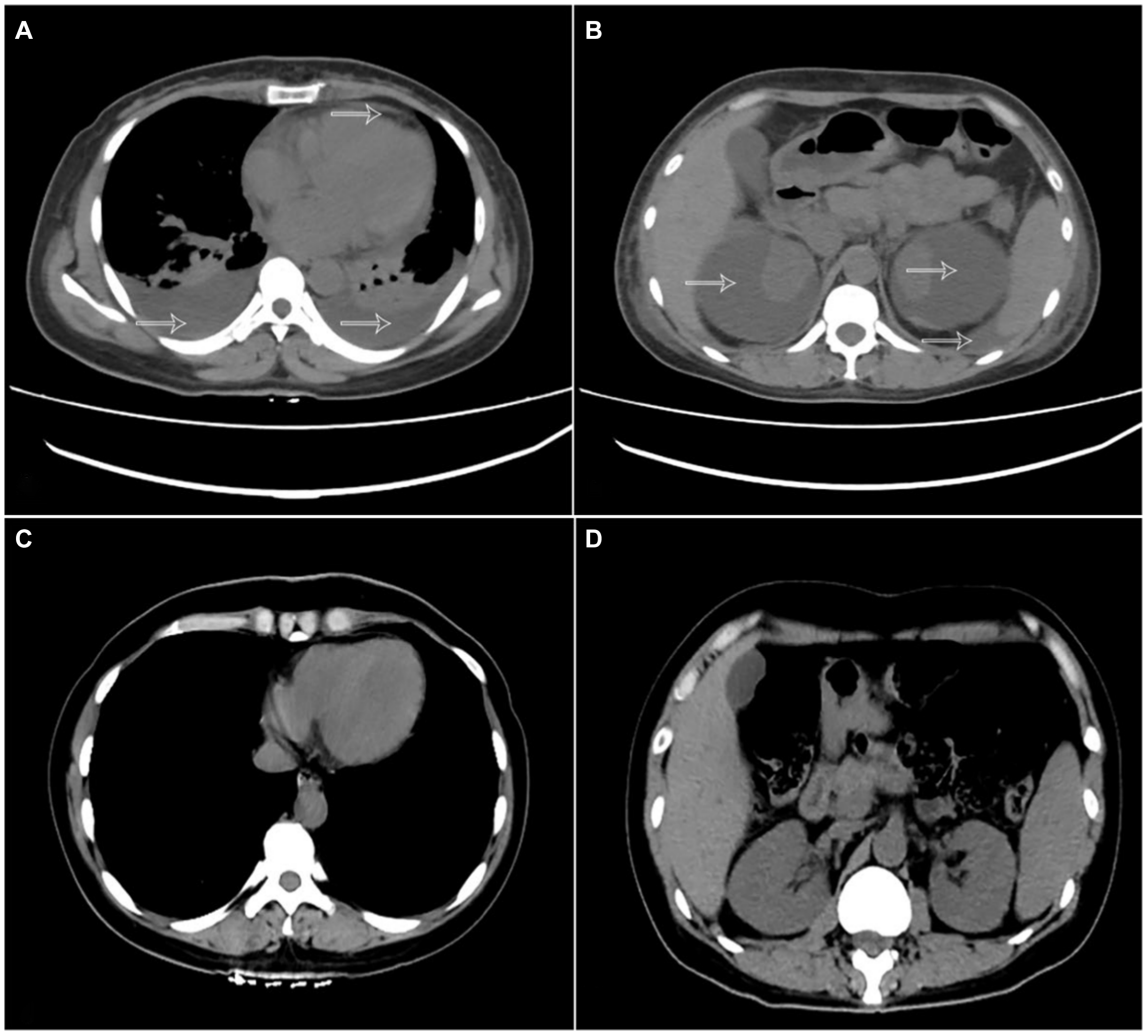
**(A)** Chest CT showing bilateral pleural effusion and pericardial effusion (arrow). **(B)** abdominal CT shows subcapsular hematoma of both kidneys and abdominal fluid (arrow). **(C,D)** CT examination of the chest and abdomen 1 week after surgery indicates pericardial effusion, bilateral pleural effusion, subcapsular hematoma of both kidneys, and abdominal effusion disappeared.

A 40-year-old woman was admitted to the hospital with bilateral lumbar and abdominal pain for 1 month, which aggravated for 4 days. The patient denied a history of trauma and surgery, a history of chronic diseases such as hypertension, diabetes, and heart disease, a history of infectious diseases such as hepatitis, tuberculosis, and typhoid fever, and a history of gastrointestinal and gynecological tumors, and no symptoms such as bone pain, butterfly erythematosus, or photosensitivity. Physical examination on admission were as follows: blood pressure 180/110 mmHg, breathing 29 times/min, unable to lie down or sit upright to breathe, painful face, anemia appearance, pale face, flat abdomen, abdominal tenderness, obvious in the left waist and umbilical circumference, no rebound pain or muscle tension, positive clicking pain in the kidney area and ureter walking area, and irregular mass in the bilateral rib abdomen. Laboratory tests indicated serum protein electrophoresis (−), anti-ANCA-GBM detection (−), urine Benchia protein characterization (−), hematuric amylase determination (−), anti-streptococcal hemolysin O (−), anti-nuclear antibody profile +ANA (−), alpha-fetoprotein (−), carbohydrate antigen 125 (−), immunoglobulin (−), and myocardial infarction (−). Laboratory examination: blood Creatinine: 375 umol/L, plasma albumin 30.1 g/L, hypersensitive C-reactive protein: 52.845 mg/L, blood routine: HB: 85 g/L, urine routine: 34 WBC/μl, 246 RBC/μl, NTproBNP: 12110 pg./mL. Chest CT (Figure 1A) showed double lung pneumonia, pulmonary edema, and a small amount of pericardial effusion. There was bilateral pleural effusion, the anteroposterior diameter of the right pleural effusion was about 23 mm, and the anteroposterior diameter of the left side was about 20 mm. Full abdominal CT (Figure 1B) showed bilateral renal subfascial effusion or chronic hematoma, right posterior 49 mm, left posterior 64 mm, and retroperitoneal, lower abdominal, and pelvic effusion or exudation.

After admission, an active intravenous infusion of human albumin increased the serum albumin level to 40 g/L, but the symptoms of multiple serosal effusions were not alleviated. The blood pressure remained between 180 and 200/110–120 mmHg for a long time. We contacted the cardiovascular department for an urgent consultation and the combined use of three antihypertensive drugs, but the blood pressure was still poorly controlled. Considering that it was related to renal hypertension, it was necessary to relieve renal compression in time, and “percutaneous bilateral perirenal fluid puncture and drainage” was performed under emergency local anesthesia after the exclusion of surgical conjunctivitis. During the operation, the needle was inserted around the left kidney, approximately 5 cm in length, the safety guide wire was placed, the leather sheath was indentured after expansion, and a 14F latex catheter was indentured to drain 1.2 L of dark-brown blood fluid with blood clots. Puncture drainage around the right kidney was performed using the same method. The puncture fluid was brown with high tension. A 14F latex catheter was placed, and 1.5 L of dark-brown blood fluid was drained. The patient was instructed to take absolute bed care within 1 week after surgery. CT examination of both kidneys 1 week after surgery (Figure 1C) showed that the subcapsular effusion of the right kidney was significantly reduced and the subcapsular effusion of the left kidney basically disappeared compared with the preoperative image. CT examination of the chest (Figure 1D) showed that the bilateral pleural effusion was significantly reduced and the pericardial effusion was basically reduced compared with the preoperative image. After the operation, the patient’s waist and abdominal pain improved significantly, his blood pressure gradually returned to normal, and his symptoms of shortness of breath and orthopnea gradually subsided, so the drainage tubes in the bilateral surgical area were removed. The patients were followed up 1 and 2 months after the operation, and both renal subcapsular effusion and polyserous effusion disappeared, and NTproBNP and creatinine returned to normal.

## Discussion

Page kidney (6) was first described by Irving Page in 1939 in a study of inducing high blood pressure in dogs by wrapping one or both kidneys in cellophane, followed by Engel and Page in 1955, in which the first clinical case of Page kidney was reported in a football player who had suffered a blunt injury to the lateral abdomen. Since 1955, more than 100 cases of Page kidney have been reported, and traumatic or iatrogenic injury has been shown to be the most common cause of Page kidney (7). Non-traumatic or spontaneous causes of Page’s kidney disease are rare and often include arteriovenous malformations, underlying tumors, ruptured cysts, vasculitis, or spontaneous renal bleeding due to glomerulonephritis. In some rare cases, Page kidney may also be idiopathic (8). Radhika Sharma et al. (9) reported a case of Page kidney secondary to acute pancreatitis complicated with pancreatic pseudocysts. Since the patient had a history of abdominal pain, blood and urine amylase tests were negative, and combined with the results of the patient’s whole abdomen CT, the diagnosis could be excluded. In this study, the patient had no clear history of trauma or renal invasive diagnosis and treatment, and routine causes were excluded. Therefore, idiopathic Page nephropathy was considered the etiology in this study.

In this case, the female patient with Page kidney was complicated by multiple serous cavity effusions (pleural, pericardial, and abdominal effusions). The patient had no symptoms such as bone pain, butterfly erythematosus, or photoallergy and denied a history of digestive tract and gynecological tumors. The results of serum calcium and phosphorus, urine Bence Jones protein, immunoglobulin, serum protein electrophoresis, anti-ANCA-GBM detection, and the ANA test of the anti-nuclear antibody spectrum were normal. Alpha-fetoprotein, carbohydrate antigen 125, carbohydrate antigen CA19-9, and other tumor markers were negative, so myelomatous nephropathy, ANCA-associated vasculitis, lupus nephropathy, and malignant tumors were excluded. When the patient underwent percutaneous bilateral perirenal fluid puncture and drainage, the fluid in the pericardium, thoracic cavity, and abdominal cavity gradually subsided, symptoms such as shortness of breath and orthopnea gradually alleviated, and NTproBNP and creatinine indicators gradually returned to normal. Therefore, the cause of this patient’s multiple serosal cavity effusion is considered to be heart failure and renal failure caused by Page kidney, which is a relatively rare phenomenon in clinical practice.

Renal ultrasonography and CT examination showed the imaging findings of renal hematoma, which can confirm the diagnosis of Page kidney. Selective renal arteriography can rule out renal vascular disease. There are no uniform guidelines for the treatment of Page’s kidney. With the medical progress of antihypertensive drugs, anti-RAAS drugs have been used for the conservative treatment of Page’s kidney (10). Most of the reported cases were mainly treated by surgery, such as nephrectomy, renal envelope resection, and percutaneous drainage (1, 11). The patient had high blood pressure at admission, and the combined use of three antihypertensive drugs was not effective. After the emergency, “percutaneous bilateral perirenal puncture and drainage” was performed to relieve renal parenchymal compression. The patient’s blood pressure gradually returned to normal, creatinine returned to normal, and the accompanying phenomenon of polyserous fluid accumulation gradually subsided. Therefore, when hypertension is still not controlled after drug treatment, surgical treatment is still a classic treatment that cannot be ignored, relieving the external pressure of renal parenchyma, correcting hypertension, and maintaining the normal function of renal parenchyma tissue.

## Conclusion

In conclusion, secondary hypertension caused by Page kidney is treatable, and ultrasound and CT scanning are quick, simple, and effective ways to diagnose this disease. When Page kidney is complicated with multiple serous effusions, the effect of simple application of antihypertensive drugs is poor, and early perirenal puncture drainage can achieve better clinical efficacy.

## Data availability statement

The datasets presented in this article are not readily available because of ethical/privacy restrictions. Requests to access the datasets should be directed to the corresponding authors.

## Ethics statement

The studies involving humans were approved by Beijing Jishuitan Hospital Guizhou Hospital Ethics Committee. The studies were conducted in accordance with the local legislation and institutional requirements. The participants provided their written informed consent to participate in this study. Written informed consent was obtained from the individual(s) for the publication of any potentially identifiable images or data included in this article.

## Author contributions

LW: Writing – original draft, Conceptualization, Formal analysis. ZQ: Writing – original draft. SZ: Writing – review & editing. GS: Methodology, Writing – review & editing. WH: Software, Writing – review & editing. ZC: Methodology, Writing – review & editing. FX: Conceptualization, Writing – review & editing. WT: Supervision, Validation, Writing – review & editing. DL: Methodology, Supervision, Validation, Writing – original draft, Writing – review & editing.
